# The spatiotemporal patterns of the beet webworm (Lepidoptera: Crambidae) in China and possible dynamics under future climate scenarios

**DOI:** 10.1093/jisesa/ieae116

**Published:** 2024-12-18

**Authors:** Jinping Zhang, Qin Yang, Zhengxue Zhao, Xiaofei Yu, Jianzhou Wei, Hua Cheng, Xuechun Zhao, Maofa Yang, Baocheng Jin

**Affiliations:** College of Animal Science, Guizhou University, Guiyang, China; Institute of Entomology, Guizhou Provincial Key Laboratory for Agricultural Pest Management of the Mountainous Region, and College of Agriculture, Guizhou University, Guiyang, China; Institute of Entomology, Guizhou Provincial Key Laboratory for Agricultural Pest Management of the Mountainous Region, and College of Agriculture, Guizhou University, Guiyang, China; Key Laboratory of High-efficiency Agricultural Plant Protection Informatization in Central Guizhou, and College of Agriculture, Anshun University, Anshun, China; Institute of Entomology, Guizhou Provincial Key Laboratory for Agricultural Pest Management of the Mountainous Region, and College of Agriculture, Guizhou University, Guiyang, China; College of Tobacco Sciences, Guizhou University, Guiyang, China; College of Science, Gansu Agricultural University, Lanzhou, China; School of Tourism, Henan Normal University, Xinxiang, China; Hydrology, Agriculture and Land Observation Group, Water Desalination and Reuse Center, Division of Biological and Environmental Sciences and Engineering, King Abdullah University of Science and Technology, Thuwal, Saudi Arabia; College of Animal Science, Guizhou University, Guiyang, China; Institute of Entomology, Guizhou Provincial Key Laboratory for Agricultural Pest Management of the Mountainous Region, and College of Agriculture, Guizhou University, Guiyang, China; College of Tobacco Sciences, Guizhou University, Guiyang, China; College of Animal Science, Guizhou University, Guiyang, China

**Keywords:** beet webworm, climate change, food security, Maxent, migratory agriculture pest

## Abstract

The beet webworm (BWW), *Loxostege sticticalis* (L.), is a notorious migratory agriculture pest of crops and fodder plants, inducing sudden outbreaks and huge losses of food and forage production. Quantifying its spatiotemporal patterns and possible dynamics under future climate scenarios may have significant implications for management policies and practices against this destructive agriculture pest. In this paper, a database containing nearly 7,000 occurrence records for the spatiotemporal distribution of BWW in China was established and its possible dynamics under future climate scenarios predicted using Maxent. We found that BWW could affect a vast geographic range of Northern China, about one third of the country’s land area. The beet webworm overwintered in most of its distribution regions. Maxent model found a northward movement and distribution reduction for BWW in China under future climate scenarios. The occurrence and overwintering regions will move northward about 0.3°N–0.9°N under warming climate scenarios, and about 40%–70% of the suitable habitat and overwintering habitat will disappear by 2100. Most of the northward movement and suitable area reduction likely will happen in 2 decades. Given the vast affected area, the abrupt outbreaks, the diverse host plants, the sensitivity to climate change, as well as their long-distance migration capacity, global scale research, and monitoring the population dynamics of BWW are essential for developing effective management strategies and mitigating its impact on agriculture and ecosystems.

## Introduction

The beet webworm (BWW), *Loxostege sticticalis* (L.) (Lepidoptera: Crambidae), is a notorious migratory agriculture pest of crops and fodder plants (Pepper 1938, Afonin et al. 2014, Frolov 2015, Huang et al. 2024). It is distributed widely throughout Europe (Frolov et al. 2008, Akhanaev et al. 2013, Hornovska et al. 2019, Muska et al. 2022, Kara et al. 2023), North America (Pepper 1938, Saunders 1938, Simmonds 1947), and Asia (Chen et al. 2016a, Enkhtur et al. 2017, Cheng et al. 2023, Mamut et al. 2023). The main plants BWW feeds on are corn, soybean, alfalfa, sugarbeet, sunflower, and grassland, but it can feed on more than a hundred plants (Kang et al. 2007, Zhang 2007, Jiang et al. 2011). The beet webworm is a migratory insect pest, and it can travel more than hundreds of kilometers within a few days under favorable wind and other climatic conditions (Zhang et al. 2008, Chen et al. 2012, Sun 2014, Huang et al. 2024), leading to sudden outbreaks and huge losses to food and forage production (Luo et al. 2009a, Frolov 2015, Chen et al. 2016a, Muska et al. 2022).

In China, there have been 3 major BWW outbreaks in 1952–1960, 1977–1986, and 2008–2009 (Zeng et al. 2018, Jiang et al. 2019, Chen et al. 2021). They have caused considerable food and forage production losses, as well as economic losses (Zhang et al. 1987, Luo et al. 1996, 2009a, b). For example, from 1979 to 1984 in Shanxi Province of Northern China, the average annual occurrence area of BWW was 0.4 × 10^5^ km^2^, leading to an economic loss of more than 50 million Yuan, despite major control efforts by the local government (Zhang et al. 1987). In 2008, more than 11 million hectares were damaged by the second-generation larva of BWW, leading to an economic loss of more than 10 billion Yuan and the most severe outbreak of BWW on record in China (Luo et al. 2009a). Although there is a history and documented records of BWW outbreaks in China, neither the plant protection departments nor any other scientists predicted the 2008–2009 outbreak (Huang 2007, Huang et al. 2008, Zhang et al. 2008a). Thus, quantifying the spatiotemporal patterns and possible dynamics of BWW under future climate scenarios may have significant implications for management policies and practices against this destructive agriculture pest.

Climate change may have important impacts on the distribution, overwintering and outbreak of BWW in China. The beet webworm is highly sensitive to climatic factors including precipitation and temperature. Moisture has a significant impact on the growth and development of BWW, and its physiological processes require sufficient water (Meng et al. 1987, Wei et al. 1987, Tang 2016). The beet webworm can tolerate very low temperatures, even up to −30°C in winter (Li et al. 2006, Akhanaev et al. 2014, Tang et al. 2017, Zhang and Jiang 2022). However, low temperatures can affect the speed of its daily developmental stages (Luo and Li 1993, Luo et al. 2016, Tang 2016), and the optimal temperature is about 18–22°C (Afonin et al. 2014, Chen et al. 2016b, Luo et al. 2016, Tang et al. 2017). Climate change will likely transform precipitation and temperature patterns, and thus the distribution of BWW at regional and global scales. Most of the previous research has found that global warming could trigger a northward expansion of the geographic ranges of many agricultural pests, as well as an increased crop loss at the global scale (Deutsch et al. 2018, Malhi et al. 2021, Skendzic et al. 2021). In the case of BWW in China, a previous study found that the overwintering regions of BWW will move northward under warming climate scenarios and the highly suitable overwintering regions will increase in area to about 1.4–2.9 times the current using Maxent modeling (Tang 2011, Tang et al. 2017). However, this study collected only 114 historical overwintering distribution records, and most were located within the central Inner Mongolia Autonomous Region. Therefore, more distribution and overwintering records are needed to clarify the impacts of climate change on the distribution and outbreaks of BWW in China.

The objectives of this paper were to (i) establish a database for the spatiotemporal patterns of BWW in China, (ii) quantify BWW spatiotemporal patterns, and (iii) predict the potential distribution patterns of BWW in China under future climate scenarios using Maxent model. We hypothesized that (i) BWW is distributed widely throughout Northern China and (ii) there may be a considerable reduction in suitable distribution areas for BWW under future global warming climate scenarios.

## Materials and Methods

### Species Distribution Data

Six thousand eight hundred and sixty-five occurrence records of BWW, *L. sticticalis* (L.) (Lepidoptera: Crambidae) in China from 1951 to 2023 were obtained from 388 scientific papers, 280 news reports, 5 books, and the Global Biodiversity Information Facility (GBIF, https://www.gbif.org/, accessed on 1 June 2024). The scientific papers were derived from visual interpretation of 6,201 English or Chinese language scientific papers downloaded from China National Knowledge Infrastructure (CNKI, https://www.cnki.net/), Weipu (http://lib.cqvip.com/), and Web of Science (https://webofscience.clarivate.cn/) using English, Chinese, and Latin names of BWW as the search “topic” or “full text.” News reports were derived from government agencies or traditional news organizations, but personal social media was excluded to avoid possible false information. The books were derived from visual interpretation of all relevant online and paper-based books from Guizhou University Library and other libraries that are available to the authors.

Each record included 3 key pieces of information: the occurrence county, occurrence date (year and month), and feeding plants. The minimum spatial resolution required for the records was at the county level (Zhao et al. 2020, 2022), so a valid record must contain an occurrence county. Occurrence date information was available for 5,372 of all occurrence records. Feeding plants information was available for 770 of all records.

Over 2,000 overwintering records were distinguished within the occurrence records. A record described as an overwintering site by its original literature was categorized as an overwintering record. Meanwhile, a record with an occurrence month from December to February was also categorized as an overwintering record based on local phenological periods in Northern China (China Meteorological Administration 2012, Cheng et al. 2023).

**Fig. 1.**
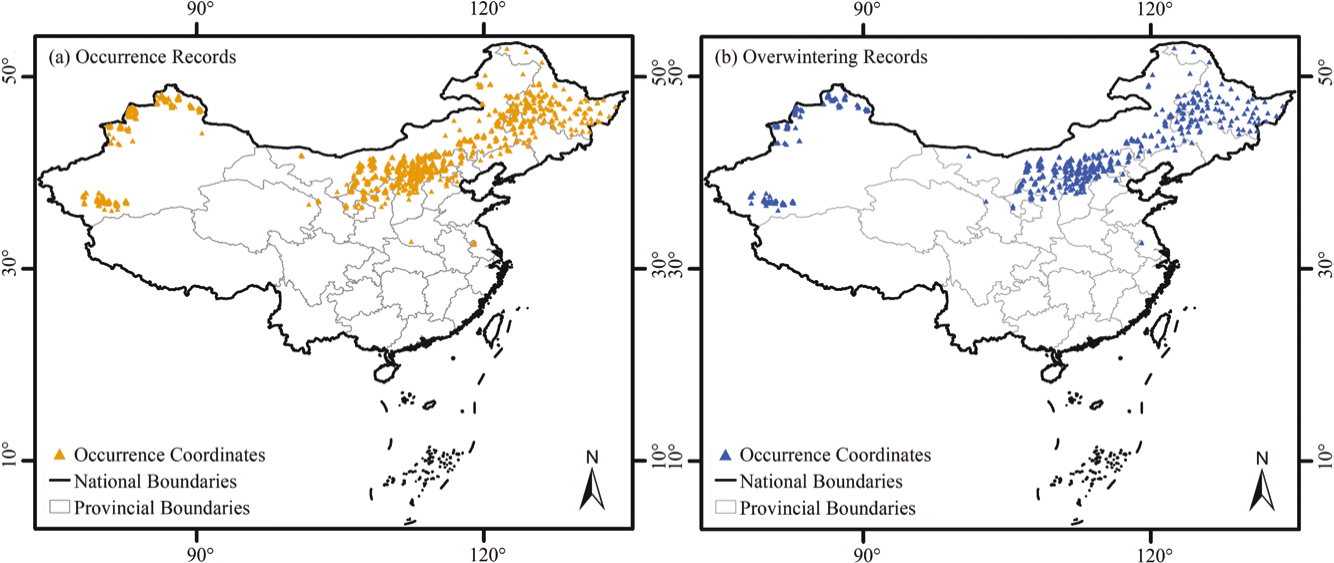
Coordinates for the occurrence (a) and overwintering (b) records of BWW in China.

Occurrence coordinates were available for 1,330 and 558 of all the occurrence records and overwintering records. Then, to avoid spatial autocorrelation, these occurrence coordinates were processed to randomly keep only one occurrence coordinate within each 2.5 arc minutes grid cell. Thus, 1,200 and 539 coordinates for the occurrence records and overwintering records were kept, respectively, and further used for the Maxent modeling (Fig. 1).

The accuracy of the database was assessed by reanalyzing 5% of the source literature. Consequently, 19 scientific papers and 15 news reports were randomly-selected and reanalyzed. The accuracies of the occurrence counties, occurrence years, occurrence months, and feeding plants were calculated separately based on the ratio of the number of correct records to the total number of reanalyzed records. Final accuracies were 98.8%, 96.3%, 96.3%, and 93.2%, respectively.

### Environmental Variables

Environmental variables included bioclimatic, crops, and grasses variables because BWW is highly sensitive to climatic influences (Pepper 1938, Afonin et al. 2014, Akhanaev et al. 2014, Kutcherov et al. 2015), as well as the availability of feeding plants (Yin et al. 2004, Jiang et al. 2011, Hornovska et al. 2019). Historical (near current, 1990–2020) and future 19 bioclimatic variables and elevation data at a resolution of 2.5 arc minutes were downloaded from the Wordclim database (https://www.worldclim.org, accessed on 1 June 2024). The historical climate data were calculated based on the monthly average minimum temperature (°C), average maximum temperature (°C), and total precipitation (mm), and downloaded from the Worldclim version 2 using the “biovars” function from the R package “dismo” (version 4.4.0, R Core Team 2024). The future environment scenarios (2041–2060, 2061–2080, and 2081–2100) provided by CMIP6 BCC-CSM 2-MR had 2 shared socio-economic pathways including SSP126 and SSP585, which respectively represented low and high emission scenarios. We also collected harvest area or distribution data for corn, soybean, sugarbeet, alfalfa, and grassland because they were the main feeding plants for BWW in China (Yin et al. 2004, Jiang et al. 2011, Hornovska et al. 2019). Corn and soybean harvest areas were sourced from the Global Pesticide Grids Dataverse (https://sedac.ciesin.columbia.edu/, accessed on 1 June 2024), while sugarbeet harvest area was sourced from the Harvard Dataverse (https://dataverse.harvard.edu/, accessed on 1 June 2024). The alfalfa harvest area was sourced from China Forage Industry Statistics from 2001 to 2010 (National Animal Husbandry Service 2017, 2018). The grassland cover percent data represented by the average from 1992 to 2022 was sourced from the Copernicus Climate Change Service (https://cds.climate.copernicus.eu/, accessed on 1 June 2024). Harvest area and distribution data were resampled to a resolution of 2.5 arc minutes to match the spatial resolution of the climatic variables. Finally, a total of 25 environmental variables possibly related to the distribution of BWW were considered in this study (Table 1).

**Table 1.** All environmental variables and their descriptions

Category	Variables	Description
Bioclimatic variables	BIO1	Annual mean temperature
	BIO2	Mean diurnal range
	BIO3	Isothermality
	BIO4	Temperature seasonality
	BIO5	Max temperature of warmest month
	BIO6	Min temperature of coldest month
	BIO7	Temperature annual range
	BIO8	Mean temperature of wettest quarter
	BIO9	Mean temperature of driest quarter
	BIO10	Mean temperature of warmest quarter
	BIO11	Mean temperature of coldest quarter
	BIO12	Annual precipitation
	BIO13	Precipitation of wettest month
	BIO14	Precipitation of driest month
	BIO15	Precipitation seasonality
	BIO16	Precipitation of wettest quarter
	BIO17	Precipitation of driest quarter
	BIO18	Precipitation of warmest quarter
	BIO19	Precipitation of coldest quarter
	Elevation	SRTM elevation data
Crops variables	Corn	Corn harvest area
	Soybean	Soybean harvest area
	Sugarbeet	Sugarbeet harvest area
Grasses variables	Alfalfa	Alfalfa harvest area
	Grassland	Average grassland cover percent from 1992–2022

### Maxent Modeling

Maxent (version 3.4.4.) model was used to model the influence of climate change on the spatial distribution of BWW in China because this model can make better predictions using fewer environmental variables and species distribution data (Phillips et al. 2006, Elith et al. 2011, Merow et al. 2013, Fourcade et al. 2014, Peach and Matthews 2020, Liang et al. 2023). To avoid multicollinearity of the variables, Pearson’s correlation was performed to test the linear correlations between the environmental variables using IBM SPSS Statistics (version 25). For any pair of highly correlated environmental variables (*r* ≥ 0.85), only one environmental variable was kept. Then, these environmental variables with inflation factor greater than 10 were also excluded. Finally, 10 environmental variables were used as Maxent modeling input variables: mean diurnal range (BIO2), mean temperature of warmest quarter (BIO10), precipitation of driest month (BIO14), precipitation seasonality (BIO15), precipitation of wettest quarter (BIO16), corn harvest area, soybean harvest area, sugarbeet harvest area, alfalfa harvest area, and grassland cover percent.

The model was optimized by choosing the feature-type combination (L, linear; Q, quadratic; H, hinge; P, product; and T, threshold) and regularization multiplier values using the ENMeval package in the R language (version 4.4.0, R Core Team 2024). We found that the optimal feature combinations were all LQHPT, and the optimal regularization multipliers for all occurrence records and overwintering records were 0.1 and 1, respectively. Other parameters of the Maxent model were as follows: “Crossvalidate” was selected as the replicated run type and 5 replications were conducted. The maximum number of background points was 10000, and the output format was “Cloglog.” The area under the curve (AUC) value was used to verify model performance (Swets 1988, Phillips and Dudik 2008, Phillips et al. 2017, Feng et al. 2021, He et al. 2021). When the AUC values of the model were greater than 0.8 and 0.9, it indicated that model performance was very good and excellent, respectively (Bogawski et al. 2019, Zhao et al. 2024). The habitat suitability values were divided into 2 levels: unsuitable and suitable based on maximum training sensitivity plus specificity Cloglog threshold (0.2875 for occurrence projection and 0.3516 for overwintering projection, Feng et al. 2021, He et al. 2021).

## Results

### Spatiotemporal Patterns of BWW

**Fig. 2.**
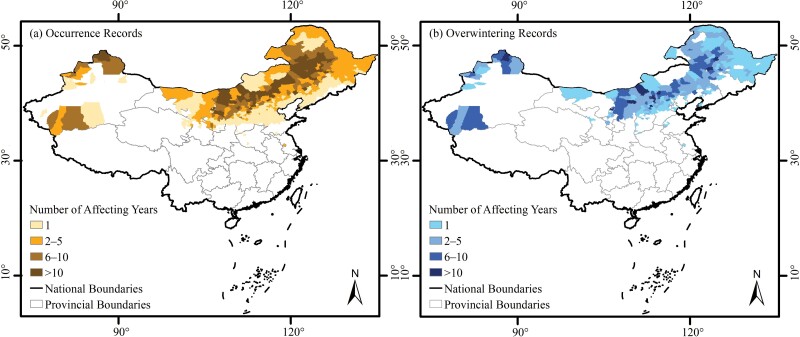
Occurrence (a) and overwintering (b) regions of BWW in China. The numbers of occurrence years for these counties without specific occurrence year information appeared as one year.

Six hundred and twenty-five counties in China were affected by BWW (Fig. 2a). These counties covered about 3.1 × 10^6^ km^2^ (or about one third) of the country’s land area, most of which was located in Northern China (Fig. 2a). The overwintering regions covered 320 counties or about 2.1 × 10^6^ km^2^ (or about one fifth) of Northern China’s land area (Fig. 2b). The most affected counties (with occurrence years greater than 10 for all occurrence records, and 6 to 10 for overwintering records) formed a narrow but continuous corridor from the center of Inner Mongolia to Northeast China (Fig. 2a and 2b). About 51.4% of the occurrence counties were also overwintering counties. For counties with more than one occurrence year, 88.5% were overwintering counties, and for the most affected counties (with more than 10 occurrence years) that percentage was 100%.

**Fig. 3.**
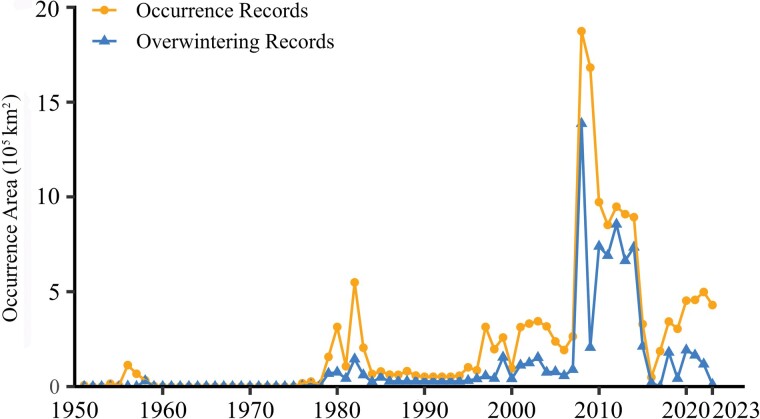
The occurrence years of BWW in China.

The areas affected by BWW exhibited a first peak around 1956, a second peak around 1982, and then a third and maximum peak in 2008 and 2009 (Fig. 3). After 2008, the areas affected were at a low level from 2009 to 2015, and then the values rebounded very quickly from 2015 to 2023 (Fig. 3). The annual overwintering regions resembled the occurrence regions (Fig. 3). Occurrence months ranged from January to December, but most of the records were from April to October.

Altogether, 143 plants, including 22 crops, 74 grasses, 27 fruits and vegetables, and 20 other plants, were affected by BWW in China. Of those, the affected percentages (the ratio between occurrence records with certain feeding plants to all 6,865 occurrence records) of corn (*Zea mays*) and grasslands were about 25% (28.2% and 24.8%). Affected percentages of alfalfa (*Medicago sativa*, 21.3%) and soybean (*Glycine max*, 20.1%) were about 20%, sugarbeet (*Beta vulgaris*), *Helianthus annuus*, *Chenopodium ficifolium* was between 10% and 20%, and 14 plants were between 1% and 10% but less than 1% for the remaining feeding plants (Table 2 and Supplementary Table S1).

**Table 2.** Main feeding plants of BWW in China. The full list of feeding plants can be found in Supplementary Table S1.

Catalogue	Name	Affected Percent (%)
Crops	*Zea mays* L.	28.2
	*Glycine max* (L.) Merr.	20.1
	*Helianthus annuus* L.	14.8
	*Triticum aestivum* L.	9.5
	*Solanum tuberosum* L.	6.8
	*Linum usitatissimum* L.	3.6
	*Brassica rapa* var. *oleifera* DC.	2.3
	*Sesamum indicum* L.	2.3
	*Sorghum bicolor* (L.) Moench	2.2
	*Fagopyrum esculentum* Moench	1.2
Grasses	Grassland	24.8
	*Medicago sativa* L.	21.3
	*Chenopodium ficifolium* Sm.	14.4
	*Salsola collina* Pall.	1.8
	*Caragana korshinskii* Kom.	1.6
	*Tagetes erecta* L.	1.6
	*Chenopodium album* L.	1.2
	Other grasses	1.7
Fruits and Vegetables	*Beta vulgaris* L.	16.0
	*Daucus carota* var. *sativus* Hoffm.	1.3
	Other fruits and vegetables	1.6

### Maxent Modeling Performance

According to the Maxent model, the mean AUC values for all occurrence records and overwintering records of 5 replicates were 0.888 and 0.905, respectively (Table 3), which indicated that the model results were very good (> 0.8) and excellent (> 0.9). For bioclimatic variables, BIO6 had the highest percent contribution for both occurrence and overwintering projections (18.7%, 19.2%), followed by BIO10 (12.2%, 13.9%), BIO2 (6.3%, 4.0%), BIO15 (4.9%, 1.9%), and BIO14 (3.3%, 1.1%). For crops, the sugarbeet harvest area had the highest percent contribution for both occurrence and overwintering projections (24.5%, 34.1%). The percent contributions of soybean harvest area and corn harvest area were less than 3%. For forage variables, the alfalfa harvest area had higher percent contributions for both occurrence and overwintering projections (23.6%, 25.0%) than grassland cover percent (0.5%, 0.2%, Table 4).

**Table 3.** AUC values of the Maxent model

Replicates	AUC	
	All occurrence records	Overwintering occurrence records
1	0.886	0.887
2	0.884	0.906
3	0.892	0.923
4	0.884	0.905
5	0.895	0.906
Mean	0.888	0.905

**Table 4.** Environmental variables and their percent contributions to predicting the distribution of BWW in China using Maxent.

Category	Variables	Percent contribution (%)	
		All occurrence records	Overwintering occurrence records
Bioclimatic variables	Precipitation of wettest quarter (BIO16)	18.7	19.2
	Mean temperature of warmest quarter (BIO10)	12.2	13.9
	Mean diurnal range (BIO2)	6.3	4.0
	Precipitation seasonality (BIO15)	4.9	1.9
	Precipitation of driest month (BIO14)	3.3	1.1
Crops variables	Sugarbeet harvest area	24.5	32.1
	Soybean harvest area	2.2	0.7
	Corn harvest area	0.9	1.9
Grasses variables	Alfalfa harvest area	23.6	25.0
	Grassland cover percent	0.5	0.2

**Fig. 4.**
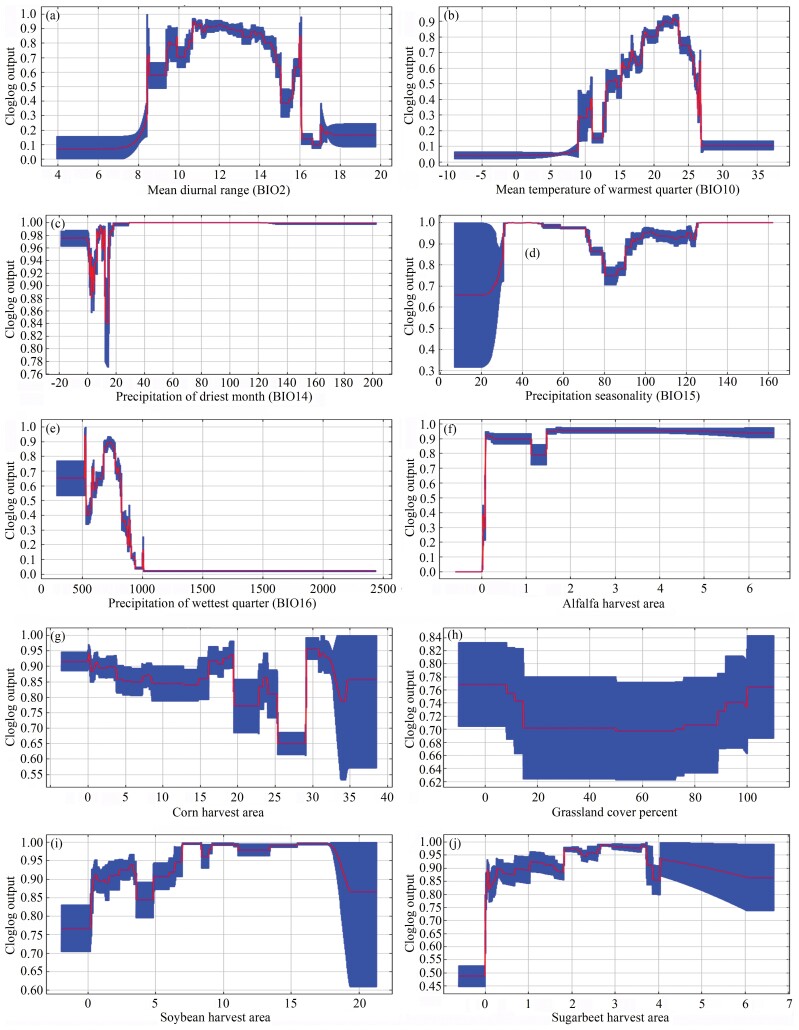
The response curve of environmental variables for the occurrence records.

**Fig. 5.**
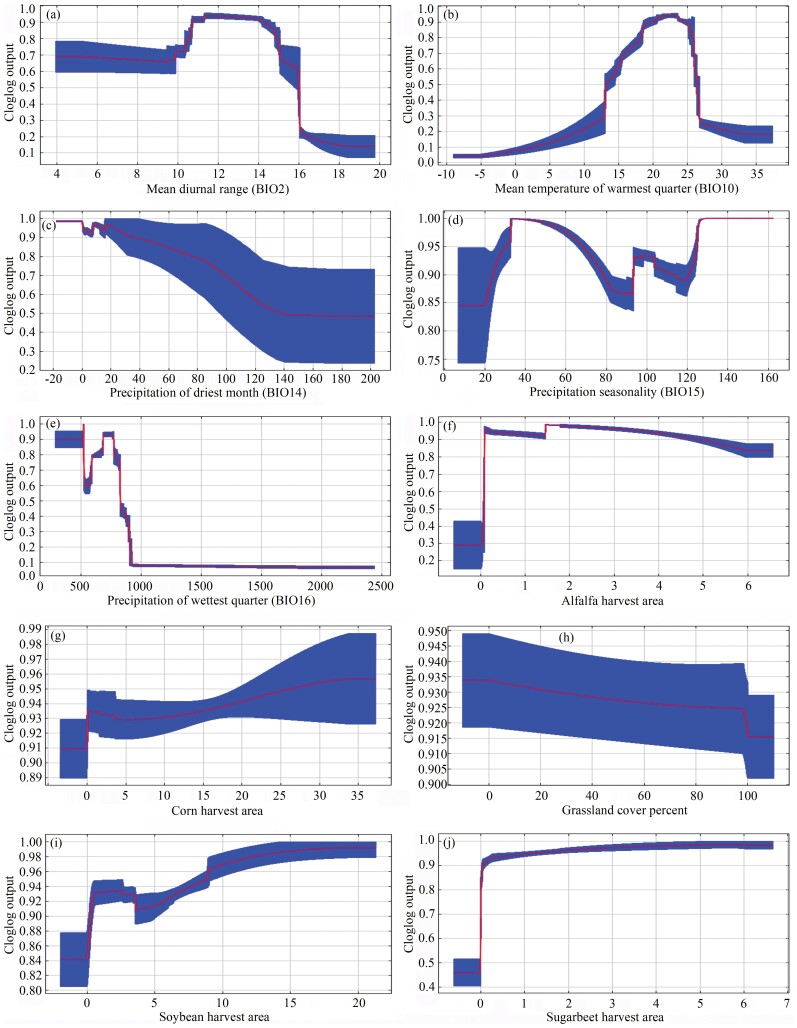
The response curve of environmental variables for the overwintering records.

These response curves revealed probability changes of BWW presence as each environmental variable changed. Ranges of each variable contributed to high probability of presence (Figs. 4 and 5). For occurrence records, the high probability occurred at 9.4°C to 16.0°C of BIO2 (Fig. 4a), 18.4°C to 26.7°C of BIO10 (Fig. 4b), 0.1 mm to 202.3 mm of BIO14 (Fig. 4c), 7.0 to 162.3 of BIO15 (Fig. 4d), 2.0 mm to 322.6 mm of BIO16 (Fig. 4e), 0.1% to 6.6% of alfalfa harvest area (Fig. 4f), 0.0% to 38.5% of corn harvest area (Fig. 4g), 0.1% to 100.0% of grassland cover percent (Fig. 4h), 0.0% to 21.3% of soybean harvest area (Fig. 4i), and 0.0% to 6.7% of sugarbeet harvest area (Fig. 4j). For overwintering records, the high probability occurred at 3.8°C to 16.0°C of BIO2 (Fig. 5a), 14.6°C to 26.0°C of BIO10 (Fig. 5b), 0.1 mm to 115.6 mm of BIO14 (Fig. 5c), 7.0 to 162.3 of BIO15 (Fig. 5d), 2.3 mm to 322.6 mm of BIO16 (Fig. 5e), 0.8% to 6.6% of alfalfa harvest area (Fig. 5f), 0.3% to 37.2% of corn harvest area (Fig. 5g), 0.1% to 100.0 % of grassland cover percent (Fig. 5h), 1.6% to 21.3% of soybean harvest area (Fig. 5i), and 0.0% to 6.7% of sugarbeet harvest area (Fig. 5j).

### Potential Distribution Patterns of BWW Under Current and Future Climate Scenarios

**Fig. 6.**
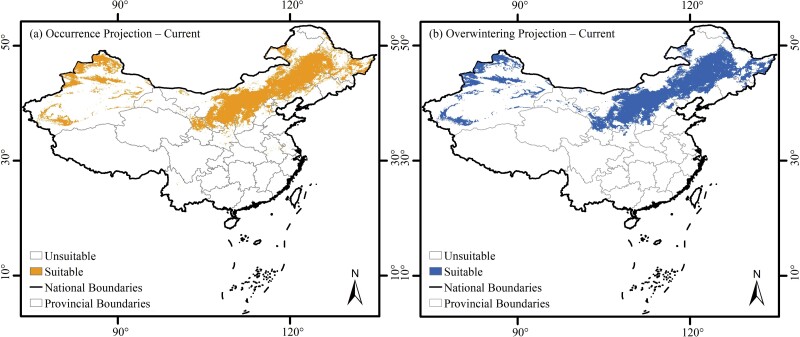
Potential distribution of BWW in China under current climate scenario.

Under current (1990–2020) climate scenario, the suitable areas of BWW for the occurrence and overwintering habitat predicted by Maxent model were concentrated in Northern China, mostly in the Northeast and Center North, but rarely Northwest China (Fig. 6). The predicted potential distribution patterns resembled their occurrence and overwintering habitats, particularly for these areas with long occurrence years.

**Fig. 7.**
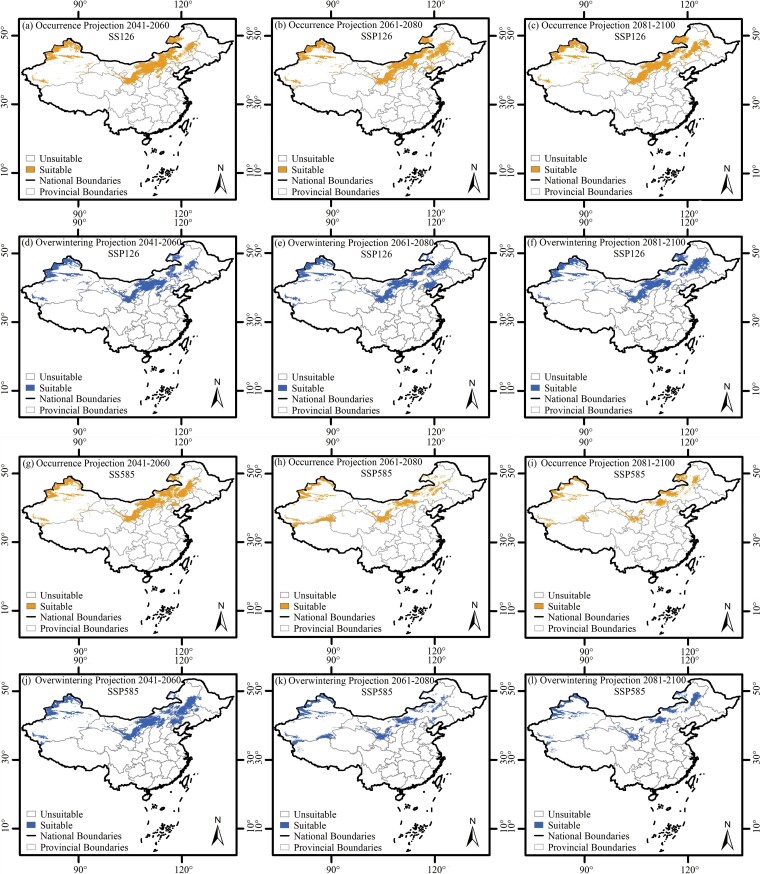
Potential distribution of BWW in China under future climate scenarios of SSP126 and SSP585 pathways from 2041–2100.

Under future (2041–2100) climate scenarios, the potential distribution for BWW occurrence and overwintering habitat predicted by Maxent were also concentrated in Northern China, but there was projected to be a large reduction in suitable area (Fig. 7). The areas of suitable habitat for all occurrence regions were projected to decrease from 14.7 × 10^5^ km^2^ (current) to 8.5 × 10^5^ km^2^ (2081–2100, SSP126 pathway), and decrease from 14.7 × 10^5^ km^2^ (current) to 4.5 × 10^5^ km^2^ (2081–2100, SSP585 pathway). Similarly, suitable area projections for overwintering regions will also decrease from 15.0 × 10^5^ km^2^ (current) to 8.1 × 10^5^ km^2^ (2081–2100, SSP126 pathway), and from 15.0 × 10^5^ km^2^ (current) to 4.3 × 10^5^ km^2^ (2081–2100, SSP585 pathway, Fig. 7).

Most suitable area reductions were projected to happen in the coming 2 decades (Table 4 and Fig. 7). For all occurrences, the area reduction percent of suitable habit was 42.2% (SSP126 pathway) and 69.3% (SSP585 pathway) from the current day to 2081–2100 climatic scenarios, but that to 2041–2060 were 42.3% and 44.3%, respectively (Table 5 and Fig. 7). Similarly, for overwintering regions, the area reduction percentages of suitable habit were 46.1% (SSP126 pathway) and 71.7% (SSP585 pathway) from the current day to 2081–2100 climatic scenarios, but for 2041–2060, they were 49.9% and 44.1%, respectively (Table 5 and Fig. 7).

**Table 5.** Potential suitable areas (km^2^) and average latitudes (°) of BWW in China under current and future climate scenarios

		2041–2060		2061–2080		2081–2100	
	Current	SSP126	SSP585	SSP126	SSP585	SSP126	SSP585
Occurrence areas	14.7 × 10^5^	8.5 × 10^5^	8.2 × 10^5^	8.6 × 10^5^	5.4 × 10^5^	8.5 × 10^5^	4.5 × 10^5^
Overwintering areas	15.0 × 10^5^	7.5 × 10^5^	8.4 × 10^5^	8.0 × 10^5^	4.9 × 10^5^	8.1 × 10^5^	4.3 × 10^5^
Occurrence latitude	42.8	42.7	42.9	43.1	41.5	43.3	42.9
Overwintering latitude	42.8	42.4	42.6	42.5	41.5	43.2	43.0

There was a northward movement of BWW under future climate scenarios (Table 5 and Fig. 7). The occurrence and overwintering regions were projected to move northward about 0.3°N–0.9°N in latitude under warming climate scenarios. The average latitudes for suitable habitat for all occurrence regions were projected to increase from 42.8°N to 43.3°N (SSP126) and from 42.8°N to 42.9°N (SSP585, Table 5 and Fig. 7). Similarly, overwintering regions were projected to increase from 42.8°N to 43.2°N (SSP126), and from 42.8°N to 43.0°N (SSP585, Table 5 and Fig. 7).

## Discussion

### Spatiotemporal Patterns of BWW in China

Spatially, the nearly 7,000 occurrence records indicated that BWW affects a vast geographic range of Northern China, covering about a third of China’s land area (Fig. 2). The wide distribution was consistent with the omnivorous nature of BWW (Kang et al. 2007, Zhang 2007, Zhang et al. 2008b, Jiang et al. 2011). In this article, we created a list of 143 plants affected by BWW in China (Table 2 and Supplementary Table S1), which can be useful for management policy making. Interestingly, although corn is the most widely planted crop in China, its affected percentage (28.2%) was only slightly higher than other feeding plants such as alfalfa (21.3%), soybean (20.1%), sugarbeet (16.0%), and sunflower (14.8%). Corn may be not a favorite feeding plant of BWW (Yin et al. 2005, Wang et al. 2015) because feeding larvae with corn leads to prolonged developmental periods and decreased fertility of moths (Tribel 1989, Yin 2001, Yin et al. 2004). Other than feeding plants, precipitation was the climatic factor with the highest percent contribution to predicting the distribution of BWW in China according to the Maxent results. This factor facilitates landing of BWW during migration (Chen 2010, Han et al. 2013, Tang 2016), and moisture can affect numerous physiological activities such as the maturation of female oocytes after copulation (Meng et al. 1987, Wei et al. 1987, Chen 2010, Han et al. 2013). The response curve showed that the optimal temperature range during the warmest quarter for BWW was 20–25°C, which was close to the optimal temperature for BWW (Afonin et al. 2014, Chen et al. 2016b, Luo et al. 2016, Tang 2016, Tang et al. 2017). The wide distribution of BWW emphasized the importance of appropriate management practices in China as well as globally.

Temporally, BWW outbreaks in China have been very abrupt. For example, in 1955 the area affected was only 0.6 × 10^5^ km^2^, but in the next year (1956), the area increased sharply to about 1.1 × 10^5^ km^2^, forming the first outbreak peak of BWW in China (Jia 1983, Chen 2010). Similarly, the affected area was only 1.1 × 10^5^ km^2^ in 1981, but in the next year (1982), the area sharply increased to about 5.5 × 10^5^ km^2^, forming the second outbreak peak (Yan 1985, Chen 2010). In the same way, in 2007 the affected area was only 2.6 × 10^5^ km^2^ with the prediction of only a moderate outbreak in 2008 (Zhang et al. 2008a). Instead, however, the affected area increased dramatically to about 18.7 × 10^5^ km^2^ in 2008, forming the most severe outbreak of BWW on record in China (Jiang et al. 2009, Luo et al. 2009a, Zhang et al. 2009). This abrupt outbreak was probably because the adult BWW is highly migratory. They can migrate to Northern China from Russia, Mongolia, and other areas within only a few days and cause catastrophic agriculture losses under favorable wind and other climatic conditions (Zhai 2001, Zhang et al. 2008b, 2009, Chen et al. 2016b, c, Huang et al. 2024). For example, the severe outbreak of second-generation larvae in 2008 was attributed to the first-generation adults originating from eastern Siberia, eastern Mongolia, and the boundary regions of China-Russia and China-Mongolia (Zhang et al. 2009, Huang et al. 2024). Given the long-distance migration of BWW within huge geographical regions of different countries, global scale research and monitoring of population dynamics, as well as bioclimatic conditions, is needed instead of domestic and small-scale monitoring to prepare for the next outbreak in China.

### Overwintering Regions of BWW in China

There are conflicting statements about the overwintering regions of BWW in China. Some previous studies have argued that they overwinter within relatively a small geographical range around the center of the Inner Mongolia Autonomous Region of Northern China (Chen and Meng 1988, Zhang et al. 2008, Tang 2011, Tang et al. 2017). For example, a field survey conducted between 2006 and 2007 found that their overwintering regions were located mainly within Siziwang Banner at the center of the Inner Mongolia Autonomous Region, a county with an area only about 2.6 × 10^4^ km^2^ (Zhang et al. 2008). In addition, another study collected 114 historical overwintering records of BWW in China (Tang 2011, Tang et al. 2017), most of which were located around the central Inner Mongolia Autonomous Region within an area about only 3.0 × 10^5^ km^2^. On the other hand, other studies have demonstrated that they can overwinter within a huge geographical range including most provinces of Northern China (Luo et al. 2009a, Zhang et al. 2009, Zeng and Jiang 2011, Zeng et al. 2016, Chen et al. 2022, Zhang et al. 2022).

Based on the over 2000 overwintering records compiled in this study, we found that although the central Inner Mongolia Autonomous Region is an important overwintering region of BWW (Chen and Meng 1988, Zhang et al. 2008, Tang 2011, Tang et al. 2017), they overwinter within a huge geographical range (Fig. 2). Their overwintering regions resembled their occurrence regions, covering 320 counties or about 2.1 × 10^6^ km^2^ (or about one fifth) of China’s land area (Fig. 2). The wide distribution of BWW overwintering regions was likely caused by the following reasons. First, previous research found that BWW overwinters by diapause development (Tauber and Tauber 1976), and they can overwinter in temperatures at least as low as −25°C to −30°C (Li et al. 2006, Tang et al. 2017). This is higher than the minimum temperature of the coldest month for most regions of Northern China. Second, the comparison between the overall occurrence and overwintering regions also revealed that most counties with occurrence records but no overwintering records were located mostly in the southern part of the potentially suitable area, but not in Northern China where there are colder winter temperatures (Fig. 2). Finally, BWW can use favorable microenvironments, such as sunny and leeward slopes, to increase their chances of overwintering success (Liu et al. 1985, Buren and Shi 1986).

### Northward Movement and Rapid Occurrence Area Reduction for BWW in China Under Future Climate Scenarios

It has been well documented that global warming could trigger a northward expansion of many agricultural pests, as well as increased crop losses (Chen et al. 2011, Deutsch et al. 2018, Guo et al. 2021, Malhi et al. 2021, Skendzic et al. 2021). In the case of BWW in China, based on the nearly 7,000 occurrence records obtained through this study, we also found that their occurrence and overwintering regions will move northward about 0.3°–0.9° under warming climate scenarios. Our findings were consistent with Tang et al. (2017), who also found that BWW overwintering regions in China will move northward under warming climate scenarios using Maxent modeling. Unlike Tang et al. (2017), however, who found that highly suitable overwintering regions of BWW in China would be about 1.4–2.9 times larger than today, we found that there may be a large reduction in suitable areas for BWW in China under future climate scenarios. Over 40%–70% of the suitable habitat for both occurrence and overwintering will disappear. More importantly, most of the suitable area reduction likely will happen in the coming 2 decades. The area reduction of suitable habitat from the current day to 2041–2060 is over 40%. The inconsistency in these 2 sets of findings may have been due to under sampling by Tang et al. (2017), who collected only 114 historical overwintering distribution records of BWW in China from 1951 to 2000, and most were located in the central Inner Mongolia Autonomous Region within an area about only 3.0 × 10^5^ km^2^. The northward movement and rapid occurrence area reduction for BWW under warming climate scenarios could facilitate the future agricultural production in Northern China. It is also possible that extreme weather associated with global warming could lead to catastrophic outbreaks of BWW in China (Luo et al. 2009b, Zhang et al. 2009, Huang et al. 2024), and this needs careful attention in the future.

Given the vast affected area, the abrupt outbreaks, the diverse host plants, the sensitivity to climate change, and their long-distance migration capacity, global scale research and monitoring for BWW population dynamics and related bioclimatic conditions is needed to prepare for the next outbreak in China.

## Supplementary Material

ieae116_suppl_Supplementary_Tables_S1
